# Effects of Ionic Liquid Content on the Electrical Properties of PVDF Films by Fused Deposition Modeling

**DOI:** 10.3390/ma17010009

**Published:** 2023-12-19

**Authors:** Runkai Zhou, Hong Yang, Lianzhong Zhao, Chun Wang, Chaoqun Peng, Richu Wang, Dou Zhang, Xiaofeng Wang

**Affiliations:** 1 School of Materials Science and Engineering, Central South University, Changsha 410083, China; z406429575@163.com (R.Z.); Y15121755466@126.com (H.Y.); pcq2005@163.com (C.P.); wrc910103@163.com (R.W.); 2 State Key Laboratory of Powder Metallurgy, Central South University, Changsha 410083, China; csuzlz@csu.edu.cn (L.Z.); chunwang0760@163.com (C.W.)

**Keywords:** PVDF, 3D printing, ionic liquids, piezoelectric materials, fused deposition modeling

## Abstract

In this study, polyvinylidene fluoride (PVDF) composite films were prepared by fused deposition modeling, and the effects of ionic liquid (IL) content on the printability, crystallization behavior, and electrical properties of melted PVDF were systematically investigated. The results show that the addition of IL increased the temperature sensitivity of melted PVDF and decreased its viscosity, while IL acted as a plasticizer to lower the melting point of PVDF and improve its FDM printability. The imidazole cations in IL had electrostatic interactions with the -CF_2_- groups in PVDF, which promoted the transformation of the nonpolar phase to the polar phase in PVDF; thus, the addition of IL was beneficial to the increase in the polar β phase. The PVDF with 20 wt.% IL contained the highest proportion of β phase content (32.59%). Moreover, the increase in polar β-phase content also increased the polarization strength of PVDF and improved its ferroelectric properties. PVDF with 10 wt.% IL had the highest residual polarization strength (16.87 μC/m^2^).

## 1. Introduction

The traditional fabrication process for piezoelectric devices is a subtractive manufacturing process that mainly uses cutting techniques, such as scribing, broaching, sawing, etching, etc. It is a complex process involving a long production cycle, high cost, and low material utilization rate. Moreover, the mechanical stress generated by the traditional process can lead to grain loss, strength degradation and other problems. Thus, the use of traditional methods to manufacture complex shapes with high-precision piezoelectric devices still presents significant technical challenges. The rise of 3D printing technology opens up a new avenue for the development of new piezoelectric materials; it can control the size and shape of devices, simplifying the manufacturing process of piezoelectric devices and reducing production costs. In the decades since the 1990s, 3D printing has experienced rapid growth and is a technology of great diversity [1]. Commonly known as fused deposition modeling (FDM), 3D printing creates a three-dimensional object through the extrusion of a molten filament in a line-into-surface fashion. Within FDM, the thermoplastic material is heated above its melting point and then deposited layer by layer onto the print table, following established model slices and pre-set trajectories [2].

Polyvinylidene fluoride (PVDF) is a semi-crystalline piezoelectric polymer with the presence of at least five crystalline phases: α, β, γ, θ, and ξ [3,4], of which α, β and γ are the three most common phases, α is a thermodynamically stable phase and is not electrically active, and the β and γ phases have extensive spontaneous polarization and good electrical activity. The increase in polar phase content improves the dielectric and ferroelectric properties of PVDF.

Studies on the fabrication of PVDF piezoelectric materials using FDM technology have been reported. Porter et al. [5] fabricated PVDF devices by applying an electric field at the front end of an FDM printer for in situ polarization of PVDF, and investigated the effects of printing angle and layer thickness on the four properties of the fabricated samples. Samples with higher β phase content were obtained through lower extrusion temperature, faster extrusion rate, and higher hot-end voltage. Momenzadeh et al. [6] investigated the feasibility of producing samples of different structures using PVDF homopolymer as a multifunctional raw material by FDM technique. Microstructural features were examined by SEM and FTIR, and tensile tests were performed according to the American Society for Testing and Materials (ASTM) standards [7]. Liu et al. [8] developed ionic liquid (IL)-assisted FDM technology of PVDF and demonstrated that the shear force provided by the FDM contributed to the directional arrangement of the dipoles, which led to the self-polarizing properties of the printed PVDF devices, and that the piezoelectric output voltage of the 3D-printed PVDF devices was 4.7 times higher than that of the planar PVDF devices. Kim et al. [9] prepared three-phase dielectric nanocomposites of PVDF, BaTiO_3_ (BT), and multi-walled carbon nanotubes (CNT) using the FDM technique. Among them, nanocomposites containing 1.7 wt.%-CNT/45 wt.%-BT/PVDF had the best combination of dielectric constant and loss characteristics (118 and 0.11 at 1 kHz), the results indicated that the FDM process for preparing PVDF composites is more likely to improve the dielectric properties compared to the conventional method. Zhang et al. [10] introduced multi-walled carbon nanotubes (MWCNTs) and IL into PVDF and developed a highly sensitive pressure sensor with a sandwich structure via FDM printing. The sensor had a high sensitivity (2.65 V kPa^−1^) and excellent stability, which was further successfully applied in gait recognition. However, there are still some difficulties in preparing PVDF composites by the fused deposition modeling method. On the one hand, due to the fact that PVDF tends to form a more stable α phase above temperatures higher than 70 °C, it is difficult for it to crystallize naturally to form a β phase, which is a great challenge for the preparation of PVDF piezoelectric materials with excellent properties. In order to enhance the β phase of PVDF, post-treatment processes, such as stretching [11,12], polarization [13], or annealing [14], are required. In addition, simultaneous polarization [5,15] and the addition of nucleating agents [16,17,18] have been applied to improve its piezoelectric properties. On the other hand, due to the high viscosity of melted PVDF, the melt is prone to clogging when it passes through the needle during the printing process, affecting the printing process and causing structural defects.

To improve the printability of melted PVDF and the dielectric and ferroelectric properties of the PVDF film, in this paper, IL is used as an additive in the FDM preparation of PVDF. IL can achieve a directional arrangement of PVDF molecular chains through ion-dipole interactions, which results in the preferential nucleation of the β phase and higher polar phase content. In addition, IL functions as a plasticizer, which improves FDM print quality by reducing the viscosity of the melted PVDF, minimizing print clogging and mitigating twisting and distortion during the PVDF molding process. In this paper, the effects of IL contents on the printability, crystallization behavior, and electrical properties of melted PVDF is investigated.

## 2. Materials and Methods

### 2.1. Materials

Analytically pure polyvinylidene fluoride (PVDF) was obtained from Solvay Group, Bellevue, MA, USA; 1-ethyl-3-methylimidazolium tetrafluoroborate ([Emim]+ [BF_4_]−) of 97% purity was obtained from Shanghai Macklin Biochemical Technology Co., Ltd., Shanghai, China; N, N-Dimethylformamide (DMF) of 99.5% purity was obtained from Shanghai Aladdin Biochemical Technology Co., Ltd., Shanghai, China.

### 2.2. Preparation of PVDF Films

**Figure 1:**
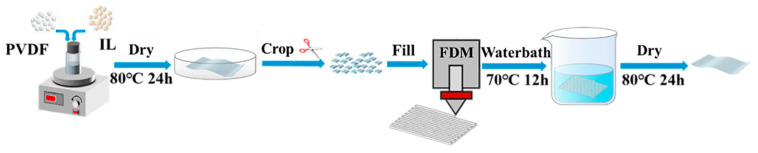
Diagram of the fabrication process of PVDF piezoelectric film by FDM.

Pure PVDF and its composite films were prepared by FDM, and the preparation process is shown schematically in Figure 1. Samples of 1-ethyl-3-methylimidazolium tetrafluoroborate ([Emim]+ [BF4]−) with mass fractions of 5 wt.%, 10 wt.%, 15 wt.%, and 20 wt.%, respectively were weighed, and mixed homogeneously with N, N-dimethylformamide (DMF) to obtain IL, and the PVDF powder was dissolved in IL to obtain the homogeneous mixed solution. The solution was poured into a petri dish and placed in a vacuum drying oven and dried at 80 °C for 24 h, until the DMF was completely volatilized and the PVDF/IL composite films were obtained.

The composite film obtained by solution casting was removed, cut into 3 cm × 3 cm samples and placed into the feed slot of the homemade 3D printer. The extrusion temperature, needle temperature, printing speed and other parameters were set in the software (REPETREL, ver:4.004), the slicing settings of the printed model were determined, the printing platform was set to zero, and the printer was then heated for 10 min under the desired printing temperature to start the printing. PVDF samples without IL were prepared by placing the PVDF powder directly into the printer slot for printing. After printing, the samples were soaked in deionized water at 70 °C for 12 h to remove the residual IL. After soaking, the samples were placed in an oven and dried at 80 °C for 24 h.

### 2.3. Polarization Process

The printed samples were polarized by corona polarization. The sample was placed directly under the needle tip electrode, and after the cavity temperature was heated to 80 °C, the voltage was slowly increased until the leakage current was shown to be 0.4 mA. This state of polarization was held for 3 h and then the heating was turned off. When the temperature had cooled to room temperature, the voltage was turned off and the sample taken out.

### 2.4. Characterization

A rotational rheometer (AR2000EX, TA, New Castle, DE, USA) was used to determine the rheological properties of PVDF and its composites in the molten state. The samples used for testing were first placed on a magnetic stirring table at 175 °C for 3 min to completely melt the sample particles. After cooling to room temperature, the samples were cut into round pieces 20 mm in diameter for testing. Rheological tests were performed using a parallel plate measuring system with a circular upper contact surface 20 mm in diameter and with 1 mm spacing between parallel plates heated by a forced convection oven attached to the rheometer. Steady-state rheological tests were performed at 180 °C with a pre-shear of 30 s at a shear rate of 10 s^−1^ ranging from 0.01 s^−1^ to 1000 s^−1^. Variable temperature rheological tests were performed at a shear rate of 1 s^−1^ in the temperature range of 180 °C to 230 °C. A field emission scanning electron microscope (SEM, Nova Nano 230, FEI, Hillsboro, OR, USA) was used to observe the surface morphology of the 3D printed samples, and an X-ray diffractometer (XRD, Smartlab SE, Rigaku, Tokyo, Japan) was used to obtain the diffraction patterns of the films at a scanning rate of 5°/min between 10° and 50°. A differential scanning calorimeter (DSC, 204F1, NETZSCH, Selb, Germany) was used for thermal analysis of the films under a nitrogen atmosphere at a temperature rise rate of 10 °C/min. A Fourier transform infrared spectroscope (FTIR, Nicolet iS50, Thermo Fisher, Waltham, MA, USA) was used to obtain the absorption spectra of the films in the range of 400–1800 cm^−1^, with 32 scans and a resolution of 4 cm^−1^. Magnetron sputtering was used to prepare samples by plating circular gold electrodes with a radius of 1 mm on the surface of the PVDF films to be tested. A precision impedance analyzer (E4990A, Keysight, Santa Rosa, CA, USA) was used to measure the dielectric properties of the films in the frequency range of 40 Hz~10 MHz, and a ferroelectric analyzer (TF analyzer 2000, aixACCT, Aachen, Germany) was used to test the electrical hysteresis loop (P-E curve) of the samples at 10 Hz frequency.

## 3. Results and Discussion

### 3.1. Melted PVDF Rheological Properties

**Figure 2:**
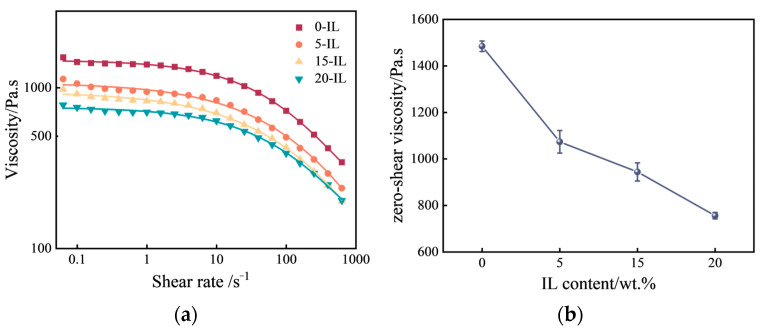
(**a**) Viscosity profiles of PVDF and its composites at 180 °C; (**b**) zero shear viscosity of PVDF melts with different IL additions.

Figure 2a shows the viscosity profiles of PVDF and its composites at 180 °C. As the shear rate increased, the viscosities of the samples all showed a decreasing trend, indicating shear-thinning flow behavior. Due to the long chain molecules of PVDF being entangled into spherical shapes at rest, under the action of shear, the globular molecular chain was oriented along the direction of shear stretching, leading to a reduction in the resistance to flow, thus reducing the viscosity at high shear rates. The viscosity curves were fitted using the Carreau–Yasuda model, which is applicable to the shear-thinning behavior of polymers [19,20]. The Carreau–Yasuda rheological equation is as follows [21]:

(1) $$\frac{\eta -\eta_{\infty }}{\eta_{0}-\eta_{\infty }}=\frac{1}{{\left[1+{\left(\lambda \gamma \right)}^{a}\right]}^{\frac{m}{a}}}$$

where $\eta_{0\;}$ is the zero-shear viscosity, also known as the first Newtonian viscosity. $\eta_{\infty }$ is the ultimate high shear viscosity, also known as the second Newtonian viscosity. η is the flow behavior index or non-Newtonian index. λ, m, and  a  are the relaxation time, the flow pattern index, and the Yasuda index, respectively.

The fitting results of zero-shear viscosity are shown in Figure 2b, with the zero-shear viscosities of melted PVDF containing 0 wt.%, 5 wt.%, 15 wt.%, and 20 wt.% of IL at 180 °C at 1484.5, 1073.8, 944.2, and 756.3 Pa·s, respectively. This shows that the addition of IL can weaken the attraction between the molecular chains of PVDF and promote molecular chain migration, thus effectively reducing the viscosity of melted PVDF and improving its printability.

**Figure 3:**
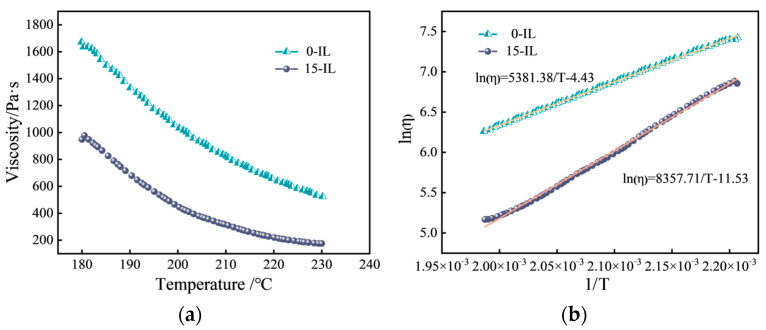
(**a**) Viscosity–temperature curves of melted PVDF with 0 wt.% and 15 wt.% IL contents; (**b**) relationship curve for ln(η)-1/T.

Figure 3a shows the variation in viscosity with temperature for melted PVDF containing 0 wt.% and 15 wt.% IL at constant shear rate (1 s^−1^). As the temperature increases, the melted PVDF softens, resulting in a sharp drop in viscosity. To study the viscosity–temperature characteristics of melted PVDF, the viscosity–temperature function is usually calculated using the Arrhenius equation, which is as follows:

(2) $$\eta =A\cdot exp\frac{E_{\eta }}{RT}$$

(3) $$\eta ln(\eta )=ln(A)+\frac{E_{\eta }}{RT}$$

where η, A, $E_{\eta }$, R, and T are the viscosity, the constant, the viscous flow activation energy, the molar gas constant, and the thermodynamic temperature, respectively. It can be shown that ln(η) is linearly correlated with  1/T, and its linear equation is fitted with the slope as $\;E_{\eta }/R$.

The fitting results are shown in Table 1. The fitted correlations were all greater than 0.995, which proved that the fitting results were good. The ln(η)-1/T relationship curve is shown in Figure 3b, which is basically a straight line, indicating that the internal microstructure of PVDF was not damaged with the decrease in temperature [22]. The viscous flow activation energy was used to characterize the temperature dependence of melted polymer viscosity, reflecting the sensitivity of viscosity to temperature changes [23]. The viscous flow activation energy of melted PVDF increased from 44.74 kJ/mol without IL to 69.46 kJ/mol with 15 wt.% IL. This showed that the addition of IL improves the temperature sensitivity of melted PVDF. Raising the same temperature, the viscosity of melted PVDF with IL decreased more sharply, which is advantageous for FDM printing of PVDF at lower temperatures.

### 3.2. Topography and Structure

**Figure 4:**
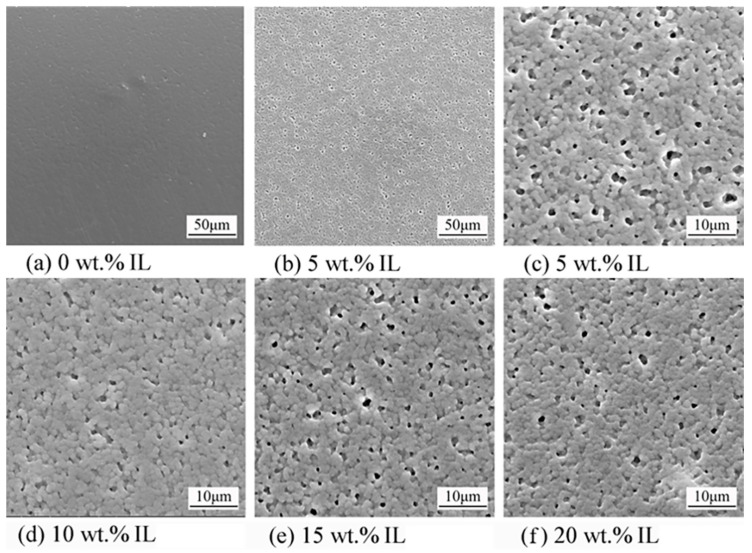
Surface SEM of PVDF/IL composites with different IL additions: (**a**) 0 wt.%; (**b**) 5 wt.%; (**c**) 5 wt.%; (**d**) 10 wt.%; (**e**) 15 wt.%; (**f**) 20 wt.%.

Figure 4 shows the SEM images of PVDF/IL composites with different IL additions. It can be observed that the surface of the PVDF film without IL was relatively flat and void free. After the addition of a small amount of IL, the surface morphology of PVDF changed significantly, and voids began to appear between the spherical crystals. Increasing the contents of IL had little effect on the change in surface morphology. 

Since the imidazole cation in IL is positively charged, and the -CF_2_- group in PVDF has a high electronegativity, the two are fully compatible in the molten state due to electrostatic interactions [24]. IL were mainly present in the amorphous region of PVDF, enriched in the interface of crystalline–amorphous regions [25], so that in the surface morphology of PVDF, it can be observed that the IL volatilized to leave a more homogeneous and finer void in the intergranular region.

The crystalline behavior of FDM-printed PVDF composites was investigated using XRD, DSC, and FTIR, and the crystalline phase was quantitatively analyzed. 

**Figure 5:**
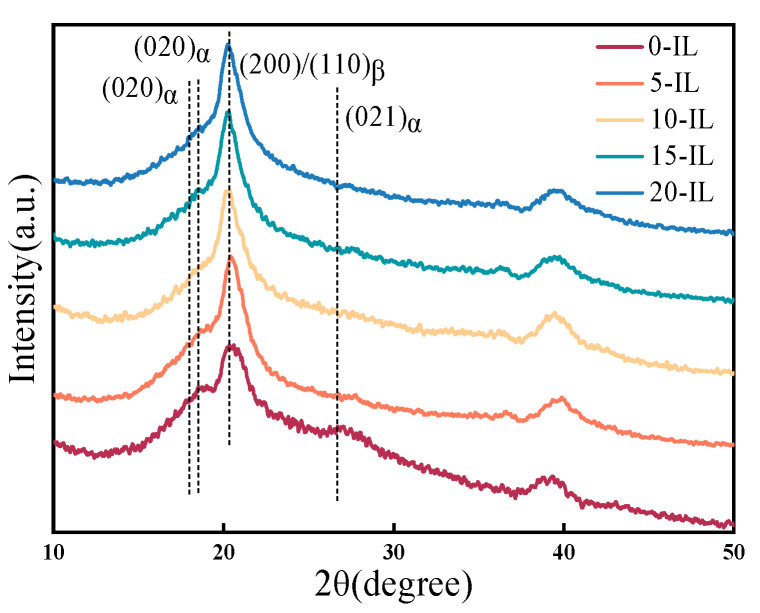
XRD diffraction patterns of PVDF materials with different IL contents.

Figure 5 shows the XRD diffraction patterns of PVDF and its composites. It can be observed that there are three characteristic diffraction peaks at 17.7°, 18.4°, and 26.6°, which correspond to the (100), (020), and (021) diffraction plane reflections of the α phase crystal surfaces in PVDF, respectively. As the IL contents increased, the intensity of the α phase diffraction peaks gradually decreased or even disappeared, while the intensity of the β phase diffraction peaks at 20.3° increased. During the melt crystallization process, there was a strong electrostatic interaction between the PVDF molecular chain and IL [26], and the -CF_2_- group was rotated under the induction of imidazole cations in IL, resulting in the transformation of the α phase into the all-trans β phase [27,28]. The addition of IL facilitated the transformation of the non-polar phase to the polar phase in PVDF. 

**Figure 6:**
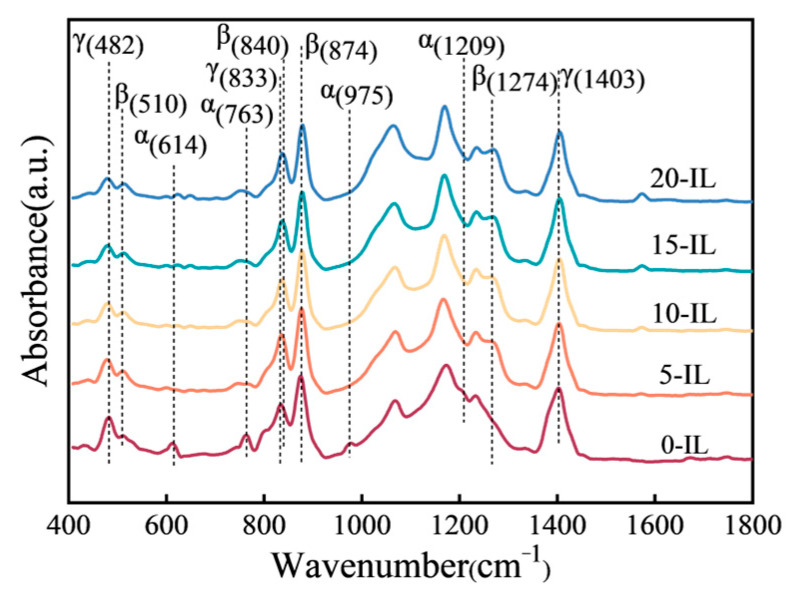
FTIR spectra of PVDF materials with different IL contents.

Figure 6 shows the infrared spectra of PVDF samples with different IL contents. It can be observed that the infrared spectra of PVDF without IL had obvious α phase characteristic absorption peaks at 614 cm^−1^, 763 cm^−1^, 975 cm^−1^, and 1209 cm^−1^, and the intensities of the absorption peaks in these four places were obviously weakened and even unobservable after the addition of IL. At the same time, after the addition of IL, the characteristic absorption peaks of the β phase appeared at 510 cm^−1^ and 1274 cm^−1^, and the intensities of the absorption peaks at 840 cm^−1^ and 874 cm^−1^ gradually increased with IL contents. In addition, the characteristic absorption peaks of the γ phase appeared in the infrared spectrum, indicating that a small amount of the γ polar phase was also present in the PVDF sample. The addition of IL promoted the transformation of the α-crystalline phase to the β-crystalline phase, which is consistent with the XRD characterization results.

Using the results of the IR spectra, the relative content of the polar crystalline phases in PVDF were calculated according to the Beer–Lambert law [29]. The equation is as follows:

(4) $$F_{\left(\beta +\gamma \right)}=\frac{A_{840}}{\frac{K_{840}}{K_{763}}A_{763}+A_{840}}$$

where $A_{763}$ and $A_{840}$ denote the intensity of the absorption peaks at 763 cm^−1^ and 840 cm^−1^, respectively, and $K_{840}/K_{763}$ is the ratio of the absorption coefficients at the corresponding wavelengths, which is 1.26 [30]. 

Since the broad peak appearing at 830–840 cm^−1^ is usually the sum of the characteristic absorption peak of the γ phase at 833 cm^−1^ and the characteristic absorption peak of the β phase at 840 cm^−1^, to separate the relative content of the β phase from the relative content of the polar phases, the relative content of the β-crystalline phase was calculated according to Equation (5):

(5) $$F_{\left(\beta \right)}=\frac{A_{840}}{\frac{K_{840}}{K_{763}}A_{763}+A_{840}}\times \frac{A_{840}}{\frac{K_{840}}{K_{833}}A_{833}+A_{840}}$$

where $A_{833}$, $A_{840}$ denote the intensity of the absorption peaks at 833 cm^−1^ and 840 cm^−1^, respectively, and $K_{840}/K_{833}$ is the ratio of the absorption coefficients at the corresponding wavelengths, which was 0.88 [31]. 

**Figure 7:**
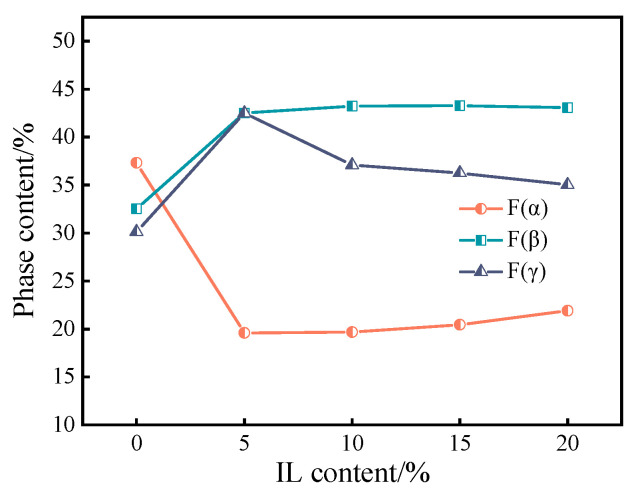
Evolution of phase content of the PVDF/IL composite material with increasing IL content.

The calculated results are shown in Figure 7. After the addition of 5 wt.% IL, the β phase content increased from 32.54% to 42.51%, and the γ phase content also increased from 30.13% to 42.51%. With further addition of IL, the content of the β phase was maintained at about 43%, while the content of the γ phase was slightly reduced but maintained at about 37%, both being higher than the 30.13% without addition. This indicates that the addition of IL to PVDF can increase the content of the β phase and the γ phase, which contributes to the formation of the polar phase. 

**Figure 8:**
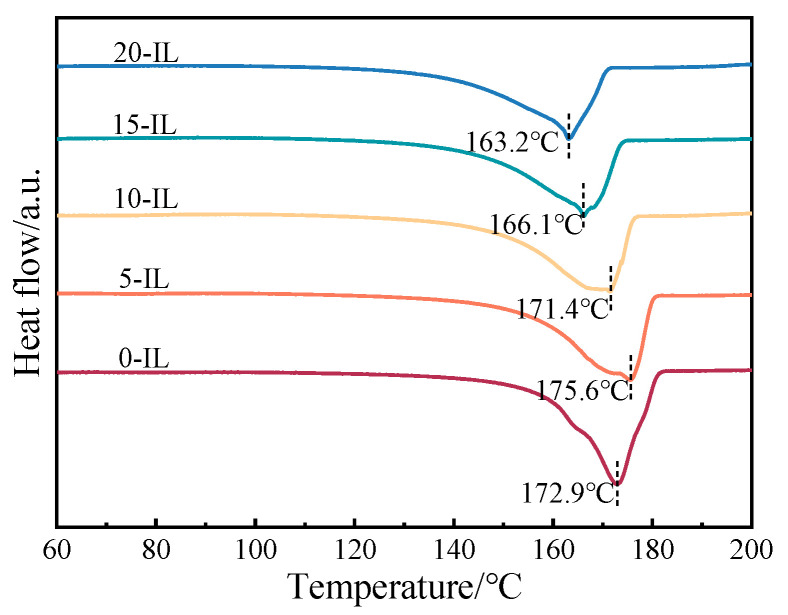
DSC curves of PVDF/IL composite material as a function of IL content.

The crystallinity of PVDF/IL composites was further calculated by DSC test analysis, and the DSC curves of PVDF with different IL additions are shown in Figure 8. The melting peak in the DSC curve was named T_m_, which was the melting point of PVDF. After the addition of IL, the value of T_m_ of PVDF increased and then decreased, and a broader melting peak was formed. The T_m_ value of PVDF without IL was 172.9 °C. After the addition of 5 wt.% IL, a large number of the β and γ phases were generated under the induced effect of IL, and since the melting point of the γ phase was 6–7 °C higher than that of the α phase [3], its T_m_ value increased to 175.6 °C. With further addition of IL, the content of the generated γ phase decreased. At this point, the polar phases in PVDF were dominated by the β phase. The β phase had a melting point similar to that of the α phase, and IL also acted as a plasticizer in PVDF [8], making it melt easily. Therefore, the value of T_m_ decreased with the further increase in IL. The decrease in melting point facilitated the realization of melted PVDF direct writing at lower temperatures.

Based on the enthalpy of the sample obtained from the test, the crystallinity of PVDF/IL was calculated by Equation (6), and then the relative content of the β phase was obtained by multiplying the relative content of the β phase by the crystallinity. The equation is as follows:

(6) $$X_{c}=\frac{\Delta H_{m}}{\Delta H_{c}}\times 100\%$$

where $\;\Delta H_{m}\;$ denotes the enthalpy measured for the actual sample and $\;\Delta H_{c}\;$ denotes the enthalpy of 100% crystallized PVDF, which is 104.5 J/g [28,32].

The calculated results are shown in Table 2. It can be observed that the crystallinity of PVDF decreased from 80.41% without IL to about 70% with IL. This can be attributed to the fact that IL, acting as a plasticizer in PVDF, increased the free volume of molecular chain segment movement and decreased the crystallization ability [28]. The decrease in crystallinity also demonstrated the good compatibility between PVDF and IL. Calculations of the β phase content showed that PVDF with 20 wt.% IL contained the highest percentage of β phase content, which was increased by 6.42%, from 26.17% without IL to 32.59% with 20 wt.% of IL. The results of the crystallographic phase analysis proved that the addition of IL can effectively increase the polar phase content.

Under the same molding process, IL has a relatively strong ability to induce polar β-phase generation compared to inorganic fillers, such as Al multi-walled carbon nanotubes (MWCNT). However, its capacity is still lower than polymethyl methacrylate (PMMA). The polar phase content comparison results are shown in Table 3.

### 3.3. Electrical Properties

**Figure 9:**
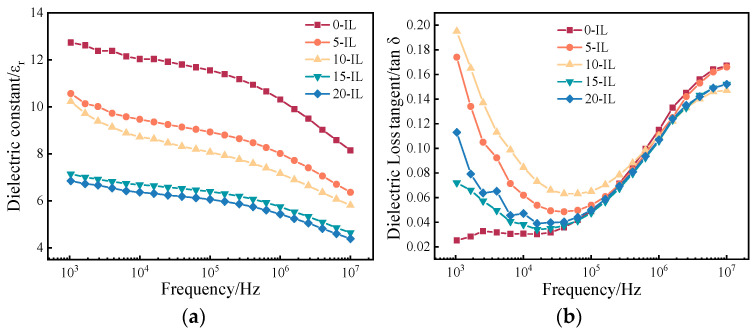
(**a**) Dielectric constant and (**b**) dielectric loss tangent of PVDF films for various ionic liquid contents from 0 to 15%.

Figure 9a,b show the variation of dielectric constant and dielectric loss of PVDF/IL composites with frequency in the frequency range of 1 kHz~10 MHz at room temperature. 

As shown in Figure 9a, the dielectric constants of the PVDF composites all showed a decreasing trend with increasing frequency from 1 kHz to 10 MHz. The dielectric polarization was dominated by ionic polarization and interfacial polarization at low frequencies and dipole polarization at high frequencies, whereas with the increase in the frequency of the external electric field, when its frequency was higher than the relaxation frequency of the PVDF, the steering motion of the dipole in the PVDF gradually lagged behind the change of the electric field, resulting in the phenomenon of dielectric relaxation [34]. At this point, dipole mobility reduction occurs, where the dipoles are not sufficiently mobile to displace as the frequency of the applied electric field exceeds the relaxation frequency [9], causing a decrease in the dielectric constant at high frequencies. In addition, the dielectric constant of PVDF/IL composites gradually decreased from 12.73 to 6.85 with the increase in IL at 1 kHz frequency. The reason for this is that the removal of the IL during the water bath treatment leaves a large number of pores in the film, the decrease in film density leading to a decrease in the dielectric constant [35]. However, at a frequency of 1 kHz, the dielectric constants of the experimentally prepared films were all greater than those of PVDF prepared by precipitation printing (3.55) reported by TU et al. [36]. In this case, PVDF with 5 wt.% IL had the highest dielectric constant and it was also higher than the dielectric constant of PVDF prepared by solution casting (9.2) [37].

As shown in Figure 9b, the dielectric loss of PVDF/IL composites was higher than that of pure PVDF materials at low frequencies, and both showed a decreasing and then increasing trend with increasing frequency. The dielectric loss was due to the dipoles inside the PVDF in the process of movement, with the energy loss causing heat energy dissipation. At low frequencies, the residual IL causes conduction losses due to increased conductivity, resulting in high dielectric losses [9,28]. When the frequency is higher than 10^4^ Hz, the conductivity loss can be ignored. This time, the increase in dielectric loss was mainly due to the existence of a dielectric relaxation phenomenon in the PVDF, which created hysteresis in the dipole flip-flop movement and produced repeated friction, resulting in high dielectric loss.

**Figure 10:**
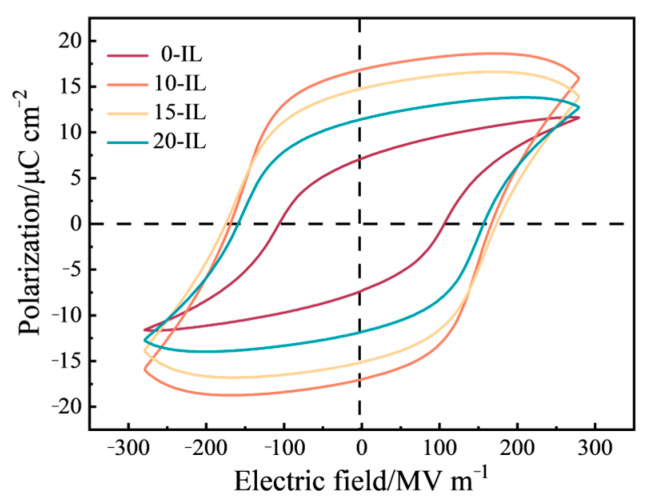
The ferroelectric hysteresis loops of PVDF composite material with different IL addition amounts under the electric field intensity of 280 MV/m.

The ferroelectric properties of the PVDF samples with 0 wt.%, 10 wt.%, 15 wt.%, and 20 wt.% IL were tested at room temperature, the hysteresis curves of the PVDF/IL composites at an external electric field strength of 280 MV/m are shown in Figure 10. With the addition of 10 wt.% IL, the saturation polarization intensity of the PVDF piezoelectric material increased from 11.62 μC/m^2^ without IL to 15.99 μC/m^2^, and the residual polarization intensity increased from 7.14 μC/m^2^ without IL to 16.87 μC/m^2^. This indicates that the addition of IL can increase the polarization strength and ferroelectric properties of PVDF piezoelectric materials. It also confirms that IL can induce the formation of a larger content of β crystalline phases in PVDF, because the increase in polar phase content is the basis for improving the ferroelectric properties of PVDF. However, by further increasing the IL addition to 20 wt.%, the saturation polarization strength and residual polarization strength showed a slight decrease, but were still higher than those of the pure PVDF piezoelectric material without IL. This can be attributed to the fact that a high percentage of IL left defects such as pores inside the material when it was removed during the water bath treatment process, which made the actual field strength lower than the apparent field strength, resulting in the decrease in polarization strength [36].

At an electric field strength of 180 MV/m, the residual polarization values of all IL-containing films prepared by the FDM process in this experiment were higher than those obtained by LIU et al. [27] at the same electric field strength (0.111 μC/cm^2^). In addition, the breakdown field strengths of the PVDF films prepared in this paper were all greater than 320 MV/m, while the breakdown field strengths reported by other 3D printing methods have been around 200 MV/m [8,36,38], a difference due to the fact that the PVDF films prepared in this paper were uniform in thickness and dense in structure, and thus could withstand larger electric field strengths.

## 4. Conclusions

In this paper, PVDF films with IL were prepared by fused deposition modeling, and the effects of IL content on printability, crystallization behavior, and electrical properties of melted PVDF were investigated. The rheological performance test result showed that melted PVDF and its composites exhibited shear-thinning flow behavior, indicating that it is a typical pseudoplastic material, which provides a rheological basis for the FDM of PVDF. The zero-shear viscosity of melted PVDF with IL gradually decreased while the activation energy of viscous flow increased, indicating that IL acting as a plasticizer improved the printability of melted PVDF. The relative *β*-phase content and crystallinity of the films were analyzed by FTIR and DSC, and the combined results of the XRD patterns showed that the content of the polar β-phase in the PVDF increased after the addition of IL. The β phase content of PVDF was increased by 6.42%, from 26.17% without IL to 32.59% with 20 wt.% of IL. Moreover, removal of IL through a water bath left holes in the PVDF film, causing a decrease in its densification and dielectric constant. The electrostatic interactions between the imidazole cations in IL and the -CF_2_- groups with strong electronegativity increased the polar phase content and ferroelectric properties. The residual polarization strength of PVDF was increased 2.36 times, from 7.14 μC/m^2^ without IL to 16.87 μC/m^2^ with 10 wt.% IL. The results of this work demonstrate the feasibility of preparing PVDF flexible electronic devices using FDM technology, and also provide new research ideas to improve their electrical properties.

## Figures and Tables

**Table 1:** Melted PVDF viscosity–temperature curve fitting parameters.

IL Content (wt.%)	$E_{\eta }/R$ (J/mol)	$E_{\eta }$ (kJ/mol)	Linearity
0	5381.38	44.74	0.99932
15	8357.71	69.46	0.99757

**Table 2:** Relative β phase content, crystallinity, and effective β phase content of PVDF composite material with different IL contents.

Sample	0-IL	5-IL	10-IL	15-IL
F(β) (%)	32.54	42.51	43.22	43.28
$X_{c}$ (%)	80.41	67.62	68.99	67.84
$X_{c}$(β) (%)	26.17	28.74	29.81	29.36

**Table 3:** Comparison of β-phase content of PVDF films with different fillers.

Filler	F(β) (%)	Methods	Year
MWCNT	30.76%	FDM	2021 [27]
Al	42%	FDM	2018 [33]
PMMA	58%	FDM	2018 [33]
IL	43.28%	FDM	This work

## Data Availability

All data are presented in the paper.
